# Antistaphylococcal activity of bacteriophage derived chimeric protein P128

**DOI:** 10.1186/1471-2180-12-41

**Published:** 2012-03-22

**Authors:** Aradhana A Vipra, Srividya Narayanamurthy Desai, Panchali Roy, Raghu Patil, Juliet Mohan Raj, Nagalakshmi Narasimhaswamy, Vivek Daniel Paul, Ravisha Chikkamadaiah, Bharathi Sriram

**Affiliations:** 1 Gangagen Biotechnologies Pvt. Ltd., No. 12, 5th Cross, Raghavendra Layout, Tumkur Road, Yeshwantpur, Bangalore 560 022, Karnataka, India; 2 Department of Molecular Genetics, University of Toronto, 1 King's College Circle, Toronto, ON, M5S 1A8, Canada; 3 Lecturer, Department of Microbiology, Melaka Manipal Medical College, Manipal Campus, Manipal 576 104, Karnataka, India

## Abstract

**Background:**

Bacterial drug resistance is one of the most significant challenges to human health today. In particular, effective antibacterial agents against methicillin-resistant *Staphylococcus aureus *(MRSA) are urgently needed. A causal relationship between nasal commensal *S. aureus *and infection has been reported. Accordingly, elimination of nasal *S. aureus *reduces the risk of infection. Enzymes that degrade bacterial cell walls show promise as antibacterial agents. Bacteriophage-encoded bacterial cell wall-degrading enzymes exhibit intrinsic bactericidal activity. P128 is a chimeric protein that combines the lethal activity of the phage tail-associated muralytic enzyme of Phage K and the staphylococcal cell wall targeting-domain (SH3b) of lysostaphin.

Here we report results of in vitro studies evaluating the susceptibility of staphylococcal strains to this novel protein.

**Results:**

Using the broth microdilution method adapted for lysostaphin, we found that P128 is effective against *S. aureus *clinical strains including MRSA, methicillin-sensitive *S. aureus *(MSSA), and a mupirocin-resistant *S. aureus*. Minimum bactericidal concentrations and minimum inhibitory concentrations of P128 (1-64 μg/mL) were similar across the 32 *S. aureus *strains tested, demonstrating its bactericidal nature.

In time-kill assays, P128 reduced colony-forming units by 99.99% within 1 h and inhibited growth up to 24 h.

In an assay simulating topical application of P128 to skin or other biological surfaces, P128 hydrogel was efficacious when layered on cells seeded on solid media. P128 hydrogel was lethal to Staphylococci recovered from nares of healthy people and treated without any processing or culturing steps, indicating its in situ efficacy. This methodology used for in vitro assessment of P128 as an agent for eradicating nasal carriage is unique.

**Conclusions:**

The novel chimeric protein P128 is a staphylococcal cell wall-degrading enzyme under development for clearance of *S. aureus *nasal colonization and MRSA infection. The protein is active against globally prevalent antibiotic-resistant clinical isolates and other clinically significant staphylococcal species including *S. epidermidis*. The P128 hydrogel formulation was bactericidal against Staphylococci including *S. aureus *recovered from the nares of 31 healthy people, demonstrating its in situ efficacy.

## Background

Antibiotic-resistant *Staphylococcus aureus *strains emerging from the community as well as hospital environments represent a global threat [1,2], requiring new approaches to control this pathogen. The anterior nare is the major reservoir of *S. aureus *in humans; 80% of the human population may be carriers [3]. A causal relationship between nasal colonization of *S. aureus *and serious infection has been established; thus, eliminating *S. aureus *nasal carriage may reduce the risk of infection [4,5]. Coagulase-negative Staphylococci (CoNS) are known commensal flora of the skin and mucous membranes and also colonize human anterior nares. Recently CoNS have been recognised as opportunistic pathogens responsible for the increasing incidence of serious nosocomial infections, mainly because of their affinity for the foreign materials used in prosthetics and indwelling devices. Immunocompromised patients, including those undergoing haemodialysis, are especially susceptible to these infections [6,7]. More than 80% of clinical CoNS strains and 30% to 40% of CoNS obtained from healthy carriers or patients from the community are resistant to methicillin [8].

Bactroban Nasal (Mupirocin ointment) has been approved for nasal clearance of *S. aureus *and significantly reduces the risk of postoperative staphylococcal infection in carriers [9]. However, mupirocin resistance has already been reported, and its use is restricted in many countries. A superior product for intranasal prophylaxis in at-risk patients is therefore an unmet medical need. New chemical entities take longer to develop, and killing by broad-spectrum antibiotics is undesirable. Current efforts are therefore focused on pathogen-specific biological entities such as peptidoglycan hydrolases [10], antibodies [11], and other antimicrobial peptides and proteins [12]. For example, lysostaphin is a bacterial peptidoglycan hydrolase that has been extensively studied for its antistaphylococcal activity in various animal models [13-15]. Bacteriophages are viruses that infect and kill bacteria and have co-evolved with bacterial defenses [16]. Bacteriophages have been used for human therapy in several Eastern European countries for decades [17]. Although they have not been used in clinical applications in Western countries, the United States Food and Drug Administration recently approved the use of bacteriophages to prevent bacterial contamination in meat [18]. In addition, bacteriophages are a good source of cell wall-degrading enzymes, which have been evaluated as antibacterial agents [19-21].

P128 is a novel chimeric protein that derives its staphylococcal cell wall-degrading enzymatic domain from the gene product, ORF56, of bacteriophage K and the cell wall-targeting domain (SH3b) from Lysostaphin (Pubmed accession no. of Lysostaphin gene: M 15686.1). We have previously reported the construction of this novel chimeric protein and assignment of its peptidoglycan hydrolase activity to the Cysteine, Histidine-dependent AmidoHydrolase/peptidase (CHAP) domain. We also demonstrated the efficacy of P128 in nasal clearance of methicillin-resistant *S. aureus *(MRSA; strain USA300) in a rodent model [22]. P128 is under development for topical indications including use against *S. aureus *nasal carriage. In this study we tested the antistaphylococcal activity of P128 by determining minimum inhibitory concentration (MIC), minimum bactericidal concentration (MBC), time-kill kinetics, and activity against Staphylococci from human nares.

## Methods

### Bacterial strains

All *S. aureus *strains used in the study are listed in Table 1. These include 30 clinical strains (27 MRSA strains and 3 MSSA strains) from the Public Health Research Institute, New Jersey and two USA 500 strains.

**Table 1:** MIC and MBC of P128 against 32 *Staphylococcus aureus *strains

**Sl. No**.	Strain	MIC (μg/mL)	MBC (μg/mL)
**1**	BK#13725	2	2
**2**	BK#9894	2	4
**3**	BK#2926 (USA100)	2	8
**4**	BK#13993	4	4
**5**	BK#14035	4	16
**6**	BK#12003	4	16
**7**	BK#13385	4	32
**8**	BK#15273	4	4
**9**	BK#14942	4	4
**10**	BK#19069 (USA300)	4	8
**11**	BK#11147	4	4
**12**	BK#15271	4	4
**13**	BK#14483	4	> 32
**14**	BK# 13387	4	8
**15**	BK#13228	8	8
**16**	BK#14935	8	16
**17**	BK# 13237	8	32
**18**	BK#14655	8	8
**19**	BK#18552	8	16
**20**	BK#9897 C (USA400)	8	8
**21**	BK#14284	16	16
**22**	BK#13180	16	16
**23**	BK#8374 (COL)	16	64
**24**	BK#11512	16	16
**25**	BK#11433	16	16
**26**	BK#13641	16	64
**27**	BK#2394	32	64
**28**	BK#9918	1	1
**29**	BK#14780	8	8
**30**	BK#15383	16	16
**31**	USA500/1	16	ND
**32**	USA500/2	64	ND
**33**	*S. carnosus*, ATCC 51365	0.5	0.5
**34**	*S. aureus*, ATCC 25923	4	4

MIC was determined using a modification of the CLSI broth microdilution method. P128 was tested at 256 to 0.125 μg/mL. *S. aureus *ATCC 25923 and *S. carnosus *ATCC 51365 were used as control strains. MBC was determined following the CLSI procedure by plating 100 μL from the MIC, MIC × 2, MIC × 4, and MIC × 8 wells on LB agar, and incubating the plates overnight at 37°C. Strains 1-30 constitute a global panel of distinct clinical isolates (MRSA, strains1-27; MSSA, strains 28-30) obtained from the Public Health Research Institute (NJ, USA); strains 31 and 32 are USA500.

### P128 expression and purification

P128 protein was cloned and expressed under the inducible T7 expression system in *E. coli *ER2566 strain. Details of cloning and design of the P128 clone-construct were reported previously (22).

To generate a purified preparation of P128 for the studies reported in this work, expression of P128 protein in *E. coli *ER2566 was induced with 1 mM IPTG, at 37^°^C for 4 h. The induced cell pellet was lysed and the protein in the supernatant was subjected to 0-50% ammonium sulphate precipitation using solid ammonium sulphate at 4^°^C. The precipitate was dialysed against 25 mM Tris HCl buffer pH 8.0, passed through an anion exchange column. The unbound fraction (flow through), containing P128 protein, was bound to a cation exchange column using 50 mM sodium acetate buffer at pH 6.0. The bound protein was eluted using a linear gradient of 0 to 0.5 M sodium chloride. Fractions containing P128 protein were extensively dialysed against saline and used for all the studies.

### MIC and MBC

The MIC was determined using a modified Clinical and Laboratory Standards Institute (CLSI) broth microdilution procedure [23]. Briefly, microtiter wells were pre-coated with 0.5% bovine serum albumin (BSA) to prevent nonspecific P128 adherence to the polystyrene plate, based on the method published for lysostaphin [24]. Two-fold dilutions of P128 were prepared in Mueller Hinton broth (MHB; Himedia) supplemented with 0.1% BSA (Sigma Aldrich), and 50 μL aliquots of the P128 dilutions (0.125-256 μg/mL) were added to the wells. Bacterial suspensions (0.5 McFarland standard) were diluted in MHB to achieve 1 × 10^6 ^colony-forming units (CFU) per mL. Then 50 μL aliquots of the cell suspension were added to wells containing P128. Plates were incubated under static conditions at 35°C for 18 h. The MIC was defined as the lowest concentration of P128 in which no visible growth was observed at the end of the incubation period. The MBC was also determined using the CLSI procedure. Briefly, 100 μL from the MIC, two times MIC (MIC × 2), four times MIC (MIC × 4), and eight times MIC (MIC × 8) wells were plated on Luria Bertani (LB) agar and incubated at 37°C overnight.

MIC of Vancomycin was determined for a panel of *S. aureus *isolates that represented the MIC range of P128 (1-64 μg/mL) using the CLSI broth microdilution method. Vancomycin was tested at concentrations of 0.125-256 μg/mL, and MICs were read manually after 24 h of incubation. MBC was also determined using the CLSI procedure. The reference strain, *S. aureus *ATCC 25923 was used for quality control of the assay, in case of both P128 and Vancomycin MIC and MBC determinations.

### Time-kill curve studies

The kinetics of P128 bactericidal activity were assessed in vitro using six *S. aureus *strains: BK#13237, BK#9894, BK#14780, BK#8374, BK#9918, and BK#19069. The cryopreserved test strains were plated on LB agar plate and incubated overnight at 37°C. Several well-isolated colonies were picked up and suspended in MHB broth; the turbidity was then adjusted to 0.5 McFarland standard (about 10^8 ^CFU/mL). The initial inoculum was prepared by inoculating 10 μL of each test bacterial suspension into 20 mL MHB supplemented with 0.1% BSA. After 1 h in a shaker incubator (37°C, 200 rpm), 2.7 mL aliquots of the culture were dispensed into four tubes, and 0.3 mL P128 was added. A 0.3 mL aliquot was immediately removed to determine the initial CFU (0 h). Incubation was continued, and 0.3 mL aliquots were taken at 1, 2, 4, 8, and 24 h. The cultures were serially diluted in sterile saline immediately after sampling and plated on MHB agar. After overnight incubation of the plates, CFU were determined. The time-kill curve was plotted based on bacterial survival at the sampling intervals [25].

### Efficacy of P128 hydrogel applied to *S. aureus *on agar surface

**Figure 1:**
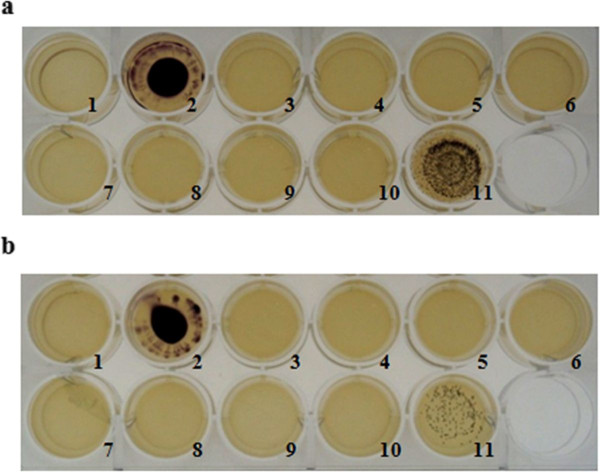
**Efficacy of P128 gel formulation applied to *S. aureus *on agar surface**. A hydrogel formulation containing P128 protein (100 to 1.56 μg/mL) was tested for bactericidal activity when applied topically on *S. aureus *strain BK#13237 cultured on LB agar: (a) 10^3 ^CFU/well, (b) 10^2 ^CFU/well. Well #1 represents the media control, and well #2 represents the cell control. In both (a) and (b), P128 gel preparations (100-1.56 μg/mL) were added to wells #3-9; P128 protein formulated in physiological saline (100 μg/mL) was added in well #10 as a positive control; buffer gel was added to well #11 as a negative control. INT dye was added to the visualize growth of the surviving bacteria.

P128 hydrogel was formulated with hydroxyethyl cellulose (0.42%), propylene glycol (0.75%), and glycerin (2.25%) as the main excipients along with P128 protein. A formulation that contained physiological saline in place of P128 (referred to as buffer gel) served as a negative control. LB agar was poured into 24-well tissue culture plates (Tarson). *S. aureus *(BK#13237) cells at 10^3 ^CFU/well (Figure 1) and 10^2 ^CFU/well (Figure 1) were seeded on LB agar in the microwells. P128 gel was diluted two-fold in buffer gel to contain P128 protein at a concentration range of 100 to 1.56 μg/mL. P128 gel preparations were applied to wells and the plates were incubated at 37°C for 18 h. At the end of incubation, 20 μL iodonitrotetrazolium chloride (INT dye; Loba Chemie) prepared in 50 mM sodium phosphate buffer, pH 7.0 (30 mg/mL) was added to the wells to visualize viable cells.

### Bactericidal activity of P128 in simulated nasal fluid

Activity of P128 was tested in a buffer that simulated the ionic composition of nasal fluid. The simulated nasal fluid (SNF) contained 0.87% NaCl, 0.088% CaCl_2_. 2H_2_0, 0.31% KCl, and 0.636% BSA [26]. The *S. aureus *COL strain was subcultured in LB medium from an overnight culture and grown at 37°C and 200 rpm until the OD_600 _reached 1.0 to 1.5 (5 × 10^8 ^CFU/mL). 100 μL of this cell suspension (5 × 10^7 ^CFU) was centrifuged at 3000 × g for 10 min and the cell pellet was suspended in 100 μL of SNF. 100 μL of P128 prepared in SNF (1.5 μg/mL) was added to the cells. As a positive control, P128 contained in physiological saline was added to cells suspended in physiological saline. After addition of P128, tubes were incubated for 1 h in a shaker incubator at 37°C, 200 rpm. Cells were then pelleted and resuspended in 1 mL LB, and 10-fold dilutions were plated on LB agar and incubated at 37°C overnight. Cells treated with SNF or saline served as untreated cell controls.

### Efficacy of P128 gel on nasal Staphylococci in their native physiological state

Nasal commensal Staphylococci of 31 healthy people were characterized and evaluated for sensitivity to P128. A dry swab (Copan Diagnostics) was inserted into each nostril, rotated six times to cover the entire mucosal surface of the anterior nare, and slowly withdrawn. The swab from one nostril of each individual was immersed in a vial containing 200 μL P128 hydrogel (40 μg/200 μL), and a swab from the other nostril was immersed in a vial containing 200 μL buffer gel (control). The vials were placed in a biosafety cabinet for 1 h at ambient temperature (about 25°C). The entire vial contents were then spread on blood agar plates and incubated overnight at 37°C. CFUs recovered were characterized in terms of colony morphology, hemolysis on blood agar, Gram stain, and a HiStaph identification kit (Himedia).

## Results and discussion

P128 is a bacteriophage derived staphylococcal cell-wall degrading enzyme. This protein is under development in our laboratory for topical therapeutic use in humans. In this study, we tested the bactericidal activity of P128 protein on globally prevalent *S. aureus *clinical strains. We assessed the biological activity of P128 using various in vitro assays and under conditions designed to simulate physiological conditions.

P128 protein preparations used in this study were of > 95% purity. The protein expressed was in the soluble form in a standard *E. coli *expression system and purified using a 2-step ion-exchange chromatography procedure [22].

### Susceptibility to P128 determined by MIC and MBC assay

Determination of MIC and MBC is a commonly used method to assess susceptibility to antimicrobial agents. We determined the MIC and MBC of P128 for a panel of 31 globally represented strains of *S. aureus *using modified CLSI methods [23]. Microtiter plate wells were pre-coated with BSA before adding P128 to minimize nonspecific adherence and loss of protein to the polypropylene surface. The MIC of P128 for the various strains of *S. aureus *ranged from 1 to 64 μg/mL (Table 1). The MIC at which 50% of the strains tested were inhibited (MIC_50_) was 8 μg/mL. The MBC of P128 across *S. aureus *strains tested also ranged from 1 to 64 μg/mL; and the MBC_50 _was found to be 16 μg/mL (Table 1).

MIC and MBC of Vancomycin were determined using the same procedure that was used in case of P128. For the reference strain, *S. aureus *ATCC 25923 MIC and MBC of Vancomycin was found be 0.5 μg/mL and 2 μg/mL respectively. These values correlate with the reported MIC and MBC of Vancomycin for this strain, validating the assay used in this work. Vancomycin was also tested on a panel of *S. aureus *strains that represented the MIC range of P128 (1 to 64 μg/mL). MIC of Vancomycin for these strains ranged from 0.5 to 1 μg/mL and MBC ranged from 1 to 4 μg/mL (Table 2).

**Table 2:** MIC and MBC of Vancomycin against a panel of *S. aureus *isolates

**Sl. No**.	Strain	Vancomycin	
		
		MIC (μg/mL)	MBC (μg/mL)
**1**	BK#9918	0.5	2
**2**	BK# 2926	1	1
**3**	BK#19069	1	4
**4**	BK#9897	1	4
**5**	BK#8374	1	4
**6**	BK#2394	1	4
**7**	USA500/2	1	4
**8**	*S. aureus*, ATCC 25923	0.5	2

MIC was determined by modified broth microdilution method following the CLSI procedure. Vancomycin test concentration was in the range of 256 to 0.125 μg/mL. *S. aureus *ATCC 25923 was used as the control strain. MBC was determined following the CLSI procedure by plating 100 μL from the MIC, MIC × 2, MIC × 4 and MIC × 8 wells on LB agar and incubating the plates at 37°C overnight. The strains used here span the MIC range of P128.

Strains 1-6 were selected from a globally represented panel of distinct, typed clinical isolates (MSSA, strain 1; MRSA, strains 2-7) obtained from The Public Health Research Institute, New Jersey, USA; strain 7 is USA500/2, and 8 is *S. aureus*, ATCC 25923

Since MIC relates to growth inhibition activity of an antimicrobial agent, MBC may be a more appropriate measure of activity of P128 which is bactericidal in action.

### Time-kill curve studies

Time-kill assays were performed in accordance with the CLSI guidelines, with a starting inoculum of 5 × 10^4 ^CFU/mL and, various multiples of the MICs. The objective of this assay was to evaluate concentration-dependent bactericidal activity. In order to find the optimal concentration required to achieve and maintain > 99.99% killing upto 24 h, sub-MIC levels were not considered. The detection limit of the time kill curve was 10 CFU/mL.

**Figure 2:**
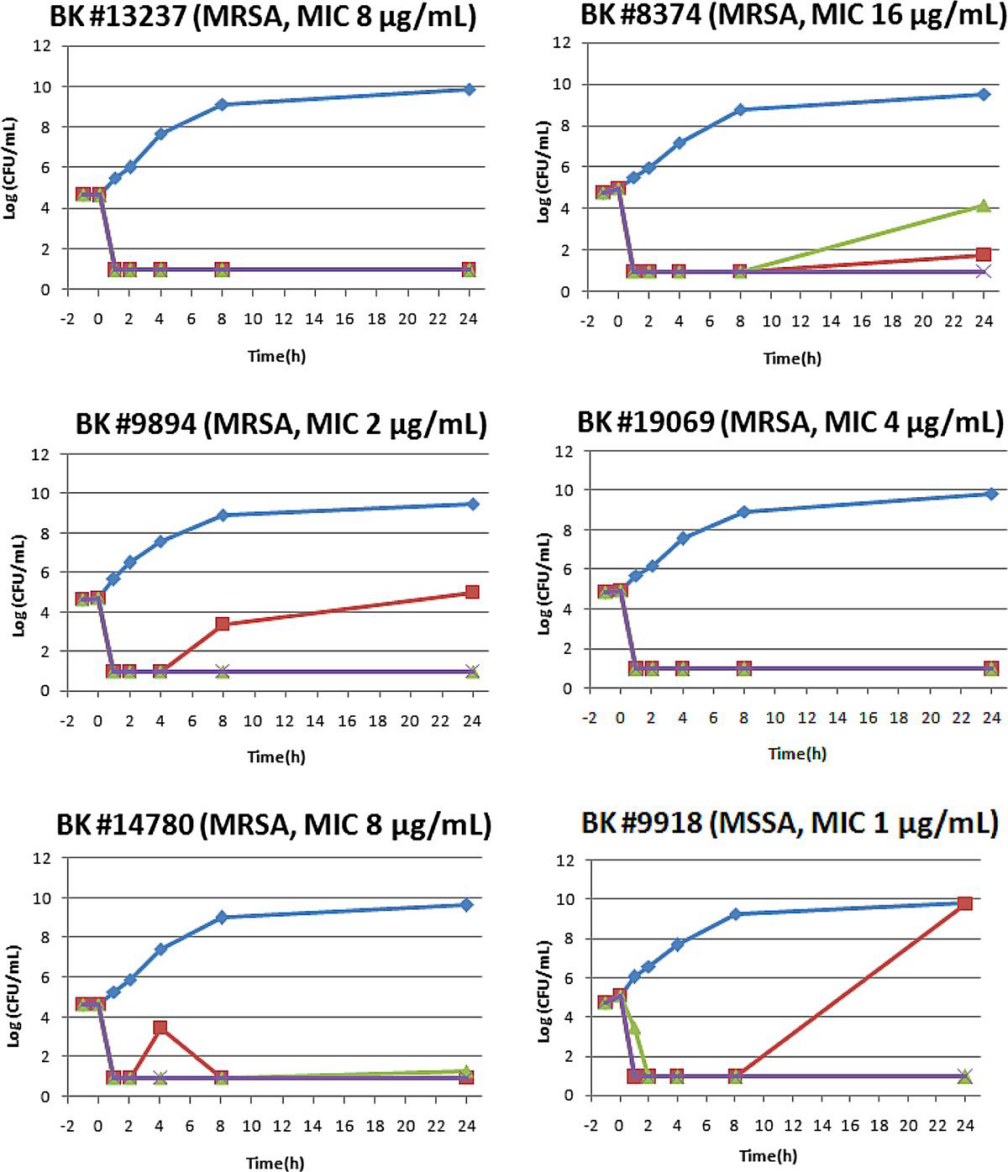
**Kill-kinetics of P128 on *S. aureus *strains**. Time-kill curves of P128 at three different concentrations (MIC, MIC × 4, and MIC × 16) on five MRSA and one MSSA strains are shown. Cell control was maintained simultaneously for each strain.

We determined the number of viable *S. aureus *cells remaining at different time intervals after adding P128 protein. Figure 2 shows the time-kill curves of P128 for six representative strains of *S. aureus*, which included five MRSA strains and one MSSA strain. P128 showed rapid, dose-dependent bactericidal activity against the MSSA and MRSA strains tested, killing of 99.99% of cells in all six strains tested within 1 h at the respective MIC concentration. At the MIC, growth was inhibited up to 24 h for all five MRSA strains and up to 8 h for the MSSA strain (BK#9918). However, the cells of BK#9918 that grew after 8 h were susceptible to P128 (data not shown). Since a concentration 4× the MIC inhibited growth of this strain for up to 24 h, we surmised that higher concentrations of P128 or repeated treatments may be required in such cases.

### Efficacy of P128 gel formulation applied to *S. aureus *on agar surface

The efficacy of P128 hydrogel was tested on solid culture medium to simulate the conditions of topical nasal application.

The assay format was designed to check availability of the protein when applied as a gel formulation. The objective was also to test efficacy of P128 gel applied to a surface where low numbers of bacterial cells are present. We have used a range of 100-1 μg/mL of protein concentration in the gel formulation. P128 gel showed complete clearance at concentrations up to 1.56 μg/mL (Figure 1).

### Bactericidal activity of P128 against *S. aureus *COL in SNF

**Figure 3:**
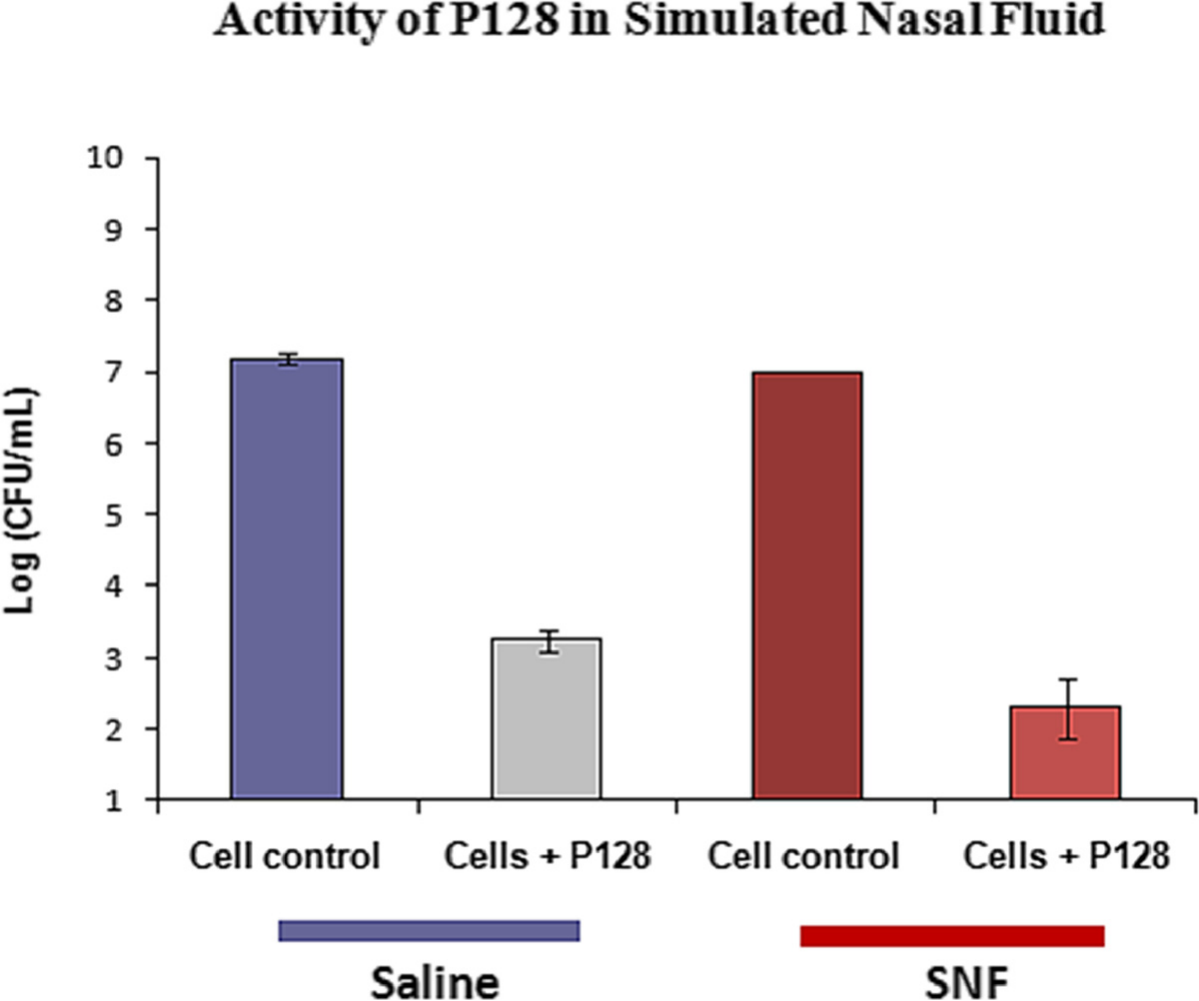
**P128 activity in simulated nasal fluid**. Bactericidal activity of P128 against *S. aureus *strain COL was tested under conditions simulating the ionic composition of human nasal fluid.

Functional efficiency and structural stability of enzymes can generally be influenced by pH, temperature, and the composition and concentrations of metal or inorganic ions in the reaction milieu. Our primary concern was that monovalent and divalent ions present in nasal fluid may have a deleterious effect on P128 activity. We therefore evaluated the activity of P128 in a composition that simulated the ionic content of normal human nasal fluid. We found that P128 reduced the staphylococcal viable count (CFU) by five orders of magnitude in SNF, comparable to the activity observed in case of P128 in physiological saline. Cells incubated in SNF that did not contain P128 were unaffected (Figure 3). These results indicate that the protein would not be influenced by the ionic content of human nasal fluid.

### Efficacy of P128 gel on nasal Staphylococci in their native physiological state

Secreted products and components such as exotoxins, exoenzymes, surface-associated adhesins, and capsular polysaccharide play a role modulating host responses to *S. aureus *infection [27]. Production of capsular polysaccharide type 5 by Staphylococci has been reported in a study using a mouse model of *S. aureus *nasal colonization [28]. The same study also showed the inability of a capsule-defective mutant to persist in mouse nares, indicating that *S. aureus *is encapsulated in the nares. The rate of methicillin resistance among CoNS isolates colonizing anterior nares of patients undergoing haemodialysis is reported to be higher than that of *S. aureus *isolates; this is accompanied by the lack of susceptibility to other classes of antibiotics [7]. Although *S. epidermidis *is responsible for most CoNS infections, other CoNS species have been associated with a variety of human diseases [6]. For example, *S. haemolyticus *is the second most commonly encountered species in clinical infections, and *S. lugdunensis *is a more recently described CoNS species [29].

In this context, we evaluated the bactericidal activity of P128 on *S. aureus *and other staphylococcal species recovered from human nares. As the first step, we characterized the nasal commensal bacteria of 31 healthy people. Speciation was carried out using the HiStaph identification kit and the *S. aureus *carriage rate was also determined. Nasal Staphylococci of 71% of the healthy people sampled consisted of CoNS species, predominantly *S. epidermidis *and *S. aureus *was found in the remaining 29% of people. Other CoNS among nasal commensal bacteria included *S. haemolyticus *and *S. lugdunensis *(Table 3). We examined nasal commensal populations in two randomly selected healthy people for comparability between the two nares with respect to bacterial load and staphylococcal species present and found both nares to be comparable (data not shown).

**Table 3:** Speciation of nasal commensal Staphylococci of healthy people

	Staphylococci recovered from healthy people	%
	**Coagulase-positive**	**29%**

2/31	*S. aureus*	6.4%
5/31	*S. aureus, S. epidermidis*	16.12%
1/31	*S. aureus, S. intermedius*	3.2%
1/31	*S. aureus, S. epidermidis*,	3.2%
	*S. haemolyticus*	
	**Coagulase-negative**	**71%**
17/31	*S. epidermidis*	54.8%
2/31	*S. lugdunensis*	6.4%
1/31	*S. delphini, S. epidermidis*	3.2%
1/31	*S. auricularis, S. epidermidis*	3.2%
1/31	*S. delphini*	3.2%

Commensal bacteria recovered from nasal swabs of 31 healthy people were plated on blood agar, enumerated, and characterized by Gram stain, coagulase test, and speciation

We then evaluated the activity of P128 hydrogel on nasal Staphylococci of 31 healthy people.

In case of nasal swabs immersed in buffer-gel, colonies were numerous, ranging from 10^3 ^- 10^5 ^CFU; estimated based on results of a preliminary experiment, where *S. aureus *cells of known CFU counts (10^3^, 10^4 ^and 10^5 ^CFU) were plated to vizualize the pattern of growth after overnight incubation of plates (data not shown). Of the swabs immersed in P128 hydrogel, 4/31 showed > 99.99% reduction in staphylococcal cell counts, 17/31 showed 99.9% reduction, 5/31 showed 99% reduction, and 5/31 showed 90% reduction (Table 4). A few colonies that grew on the plate containing P128 were found to be sensitive to the protein when tested, and hence apparently escaped the protein action.

**Table 4:** Efficacy of P128 gel on nasal Staphylococci in their native physiological state

**Volunteer No**.	CFU count		Reduction in CFU (%)
	Buffer gel	P128 gel	
1	~10^5^	16	99.99
2	~10^5^	10	99.99
3	~10^5^	18	99.99
4	15	0	> 99.99
5	~10^5^	150	99.90
6	> 10^5^	143	99.90
7	~10^5^	212	99.90
8	~10^4^	57	99.90
9	~10^4^	15	99.90
10	~10^4^	13	99.90
11	~10^4^	14	99.90
12	~10^4^	44	99.90
13	~10^4^	57	99.90
14	> 10^4^	86	99.90
15	~10^4^	29	99.90
16	~10^4^	10	99.90
17	~10^4^	64	99.90
18	~10^3^	3	99.90
19	~10^3^	2	99.90
20	~10^3^	3	99.90
21	~10^3^	6	99.90
22	> 10^5^	1200	99.00
23	~10^4^	128	99.00
24	~10^4^	220	99.00
25	~10^3^	24	99.00
26	~10^3^	22	99.00
27	~10^3^	190	90.00
28	~10^3^	110	90.00
29	~10^3^	310	90.00
30	278	17	90.00
31	250	22	90.00

Both nares of each individual were swabbed. One swab was immersed in P128 hydrogel, and the other was immersed in buffer gel (control). Staphylococcal CFU counts of nasal swabs immersed in P128 gel were significantly lower than CFU counts of control swabs

This finding shows that P128 is bactericidal to nasal staphylococcal isolates. However, we did not evaluate the presence of capsular polysaccharides, which may be assessed in future studies in our laboratory. It is important to note that the cells were treated with P128 hydrogel immediately after isolation (i.e., without exposure to any other medium or subjection to any steps of cultivation). We conclude that both *S. aureus *and CoNS are susceptible to P128 in the physiological state relevant to nasal carriage. Considering the pathogenic potential and multidrug resistance of these species, it is significant that these species were fully sensitive to P128. Further studies are needed to determine the MIC and MBC of P128 on CoNS.

Reports point to the endogenous origin of most infective *S. aureus *isolates and MRSA carriage poses an increased risk for invasive infections compared with MSSA carriage [30,31]. The worldwide spread of MRSA strains, which are often multidrug-resistant [32], combined with limited therapeutic options necessitates new approaches to combat this pathogen. Recent findings emphasize that commensal CoNS strains are also potential threats [33]. Therefore an antibacterial agent, exemplified by P128, which can target antibiotic resistant *S. aureus *as well as other clinically significant Staphylococci would meet the current medical need and warrants further development.

## Conclusions

This report describes the development and in vitro biological characterization of a chimeric antistaphylococcal protein designated P128, which exhibits rapid and selective antibacterial activity at low MIC values against a broad range of staphylococcal species, including numerous clinically relevant *S. aureus *strains.

The MIC and MBC of P128 on a global panel of clinical isolates ranged from 0.5 to 64 μg/mL. P128 showed in vitro microbicidal activity against a wide variety of staphylococcal species, including many clinically relevant *S. aureus *strains. With an MBC_50 _of 16 μg/mL, the protein was bactericidal against every *S. aureus *strain tested. P128 time-kill kinetics were determined at MIC and higher concentrations on select isolates, and P128 was found to rapidly reduce cell numbers by 99.99%. To develop P128 as a treatment to eliminate human nasal carriage, P128 was formulated as a hydrogel and tested on nasal Staphylococci recovered from healthy people. The protein was able to kill *S. aureus *under conditions representing physiological conditions. Taken together, our findings demonstrate that P128 exhibits excellent antistaphylococcal properties and warrants development for therapeutic use.

## Competing interests

The authors declare that they have no competing interests.

## Authors' contributions

BS and AV participated in the study design and coordination and data interpretation. AV, SD, PR and RP evaluated the efficacy of P128 gel in nasal Staphylococci experiments. JR, RP, PR, SD, and NN performed P128 MIC and MBC assays. JR and PR performed time-kill curve experiment. VP tested P128 activity in SNF, and RC evaluated the efficacy of P128 hydrogel in the agar surface assays. AV also helped draft the manuscript. All authors read and approved the final manuscript.
